# Indoxacarb poisoning causing methemoglobinemia treated with parenteral vitamin C: a case report

**DOI:** 10.1186/s13256-024-04455-w

**Published:** 2024-03-17

**Authors:** Lokesh Koumar Sivanandam, H. Arunkumar, Pranay Marlecha, Varsha Madamanchi, Chanchal Maheshwari, Mohammed Quader Naseer, Vivek Sanker, Tirth Dave

**Affiliations:** 1 Sri Lakshmi Narayana Institute of Medical Sciences, Puducherry, India; 2 Team Erevnites, Puducherry, India; 3 Junior Resident, Medicine, Sri Lakshmi Narayana Institute of Medical Sciences, Puducherry, India; 4 Kempegowda Institute of Medical Sciences, Bangalore, Karnataka India; 5 Maharaja’s Institute of Medical Sciences, Vizianagaram, Andhra Pradesh India; 6 Karachi Medical and Dental College, Karachi, Pakistan; 7 Ayaan Institute of Medical Sciences, Moinabad, Telangana India; 8 Noorul Islam Institute of Medical Sciences, Trivandrum, India; 9 Bukovinian State Medical University, Chernivtsi, Ukraine

**Keywords:** Indoxacarb poisoning, Insecticide, Methemoglobinemia, Ascorbic acid, Vitamin C

## Abstract

**Introduction:**

This case study reports on a suicide attempt involving indoxacarb and vitamin C. Indoxacarb is a neurotoxic insecticide used in agriculture and as a flea controller in pets. Cotton, vegetables, and fruits are treated with indoxacarb, an insecticide that can be applied both indoors and outdoors. It causes skin allergies, methemoglobinemia, and hemolytic anemia. It is also attributed to allergic reactions through ingestion, inhalation, physical contact, and translaminar action. This case report highlights use of vitamin C in methemoglobinemia caused by indoxacarb poisoning. Indoxacarb poisoning has the potential to be extremely serious and even lethal. In this instance, the patient initially had no symptoms after ingesting a substance containing indoxacarb in an attempt at suicide. However, further tests revealed methemoglobinemia and low oxygen levels.

**Case presentation:**

A 28-year-old south-east Asian female patient ingested an insecticide containing 5.25% novaluron, 4.5% indoxacarb, and 25% thiamethoxam, and reported that she noticed muddy brown urine but presented with no active signs or symptoms of poisoning. Upon examination, the patient was fully conscious, alert, and hemodynamically stable, but had an oxygen saturation of 84%. Gastric lavage was performed, and blood investigations revealed a muddy-brown-colored blood sample and methemoglobin levels of 12%. The patient was treated with high-dose vitamin C and showed significant improvement, with a drop in methemoglobin levels to 1.2% and an increase in oxygen saturation to 97%.

**Discussion:**

Indoxacarb poisoning can cause severe methemoglobinemia. Vitamin C may be a useful treatment option for methemoglobinemia caused by indoxacarb, particularly in cases in which traditional treatment with methylene blue is contraindicated or not tolerated. Hence high doses of ascorbic acid, that is, vitamin C, were administered to the patient, which lowered their methemoglobin levels and improved oxygen levels without much safety concerns.

**Conclusion:**

This example emphasizes the significance of early indoxacarb poisoning detection and treatment as well as the possible advantages of utilizing ascorbic acid in the management of methemoglobinemia, and highlights the use of vitamin C in the treatment of methemoglobinemia caused by indoxacarb poisoning. Therefore, it is important for healthcare professionals to be aware of the potential for indoxacarb to cause methemoglobinemia and to consider vitamin C as a treatment option.

## Introduction

Indoxacarb is an insecticide for exterior or interior use, used in cotton, vegetables, and fruits. It is also used as flea controller in dogs and cats [1]. Indoxacarb is a neurotoxin that acts by blocking sodium channels in the nervous system and causes methemoglobinemia, hemolytic anemia, and allergic reaction to skin (contact dermatitis) [2]. Indoxacarb poisoning can occur through ingestion, inhalation, physical contact, translaminar action, during preening, and at rewetting of surfaces [3]. In our case, poisoning occurred following ingestion of approximately 50 ml indoxacarb in a suicidal attempt due to acute financial crisis with no history of any mental health disorder. This case report highlights the use of vitamin C (ascorbic acid) in methemoglobinemia caused by indoxacarb poisoning.

## Case presentation

**Fig. 1:**
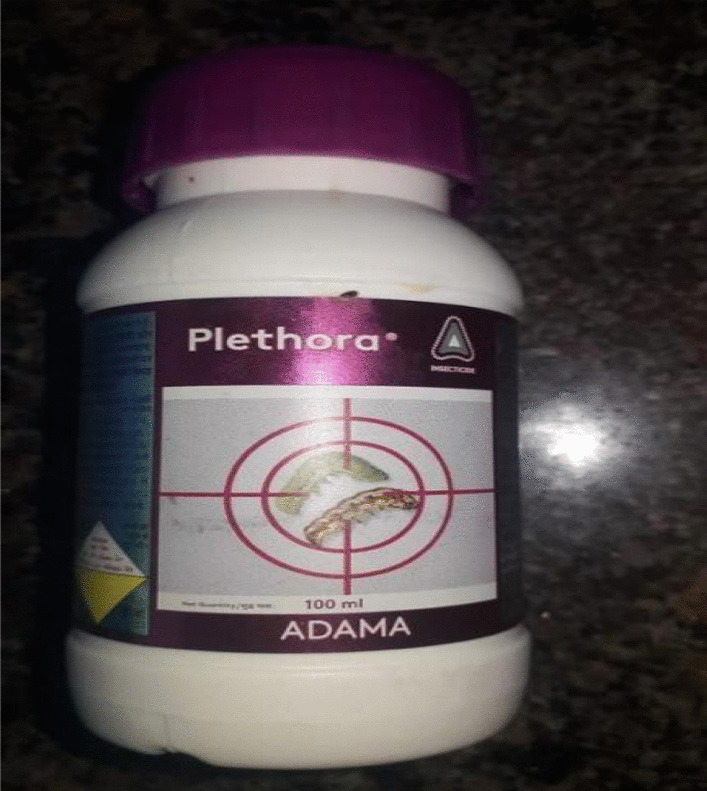
Plethora: the insecticide consumed (novaluron 5.25% + indoxacarb 4.5% + thiamethoxam 25% weight for weight, w/w)

A 28-year-old south-east Asian female patient, presented with the complaints of ingestion of an insecticide with no active signs or symptoms of poisoning after a suicide attempt. After the initial workup and verification was completed, it was found that the compound that she consumed was approximately 50 ml of Plethora and Eksona (Fig. 1), with a chemical composition of 5.25% novaluron, 4.5% indoxacarb, 25% thiamethoxam. She was married for 3 years and had a daughter. She had no history of any drug abuse or alcohol intake.

The patient reported muddy brown urine but did not report any associated nausea, vomiting, or any change in usual activities after insecticide consumption, and she did not seek any first aid. Upon examination, the patient was fully conscious, alert to time, place, and person, hemodynamically stable, and coherent with intact pupillary reflex. Gross motor examination was also unremarkable. However, her oxygen saturation was at 84% room air. Therefore, because of the significant drop in oxygen saturation, arterial blood gas (ABG) was carried out with background oxygen therapy: oxygen by face mask at 4 l/minute to maintain saturation of 95%. Education-corrected Mini-Mental State Examination (MMSE) was performed by the attending psychiatrist, and the patient scored 27/30, which was interpreted as normal. This case report has been reported in line with the Surgical CAse REport (SCARE) criteria [4].

**Fig. 2:**
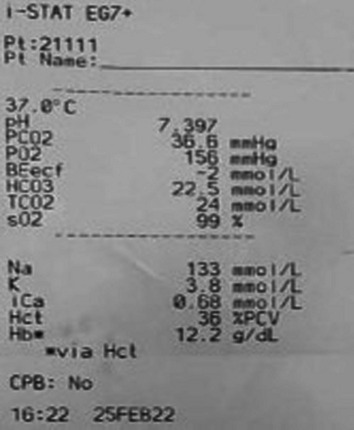
Arterial blood gas report, with oxygen saturation within normal range but pulse oximetry showing 88%

As one of the components of the compound belongs to organophosphorus compound (OPC), gastric lavage was immediately done to prevent OPC associated cardiac complication; likewise, salivation, lacrimation, urination, defecation, gastrointestinal upset, and emesis were ruled out. Blood investigations revealed muddy-brown-colored blood sample, raising the suspicion of methemoglobinemia. It was confirmed by ABG analysis, revealing oxygen saturation (SaO2) of 99% (Fig. 2), and hence the saturation gap. Methemoglobin levels upon assessment were found to be 12%, whereas normal is below 1% in a healthy individual. Furthermore, the baseline electrocardiogram (ECG) showed normal sinus rhythm. Complete blood count, renal function test, liver function test, and serum cholinesterase levels were in the normal range, indicating no significant hemolysis, hence, glucose-6-phosphate dehydrogenase (G6PD)-induced hemolysis was ruled out, but prime suspect of muddy brown color of blood and urine samples was a big question.

Upon various discussions and reviews, it was considered that the compound indoxacarb in poison has the potential of causing severe methemoglobinemia that usually symptomatically presents as orange-colored urine. Considering it as a case of poison induced methemoglobinemia, vitamin C, and methylene blue being thought of as management. In our patient, to be on safer side, we started her parenteral vitamin C, total dose 2 g/day.

First dose: 1 g in 500 ml of dextrose (5%) over 4 hours given and the next dose thereafter.

Total volume of dextrose administered: 1 l.

Total ascorbate injected: 2 g.

Shortly, significant improvement was noticed in our patient, with a gradual rise in oxygen saturation from 84% to 97% over a period of 2–4 hours. A significant clinical improvement in terms of hypoxemia was noted at the end of 4 hours and improvement in saturation gap was noted after treatment completion (after 8 hours). Repeat methemoglobin levels on assessment showed a massive drop from 12% to 1.2% [3]. The in-hospital psychiatry team assessed the factors in and around her suicidal motive and attempt, and initiated her on interpersonal counseling sessions that involved both the patient and her partner. The patient was advised to stay connected with her loved ones and safeguarding concerns against husband was evaluated, suicide prevention life line numbers and brochures were given to her, and her triggers were addressed by liaising with social support team.

She recovered soon successfully and was discharged home on the second day of hospital admission (Table 1).

**Table 1:** Tabulated Arterial Blood Gas reports on admission and after treatment

ABG	On admission	After treatment
1. pH	7.39	7.36
2. pCO2	36.6	35.6
3. HCO3	22.5	24.5
4. pO2	156	88
5. AG	10	8
6. SO2	99%	99%
7. MethHb	12%	1.2%

*pCO2* Partial pressure of carbon dioxide; *pO2* Partial pressure of oxygen; *AG* Anion gap; *SO2* Oxygen saturation; *MethHb* Methemoglobin

## Discussion

Indoxacarb is a broad spectrum (exterior or interior use [1]) insecticide that can control many insects, including the cotton bollworm and native budworm, and thus it can be used for a wide range of crops, including cotton and soybeans [5]. It is considered a “reduced-risk” pesticide that replaces organophosphate pesticides. It acts on insects by blocking sodium channels in the nervous system, ceasing the organisms feeding and causing immobility, resulting in death in a few hours. It also exerts human toxicity characterized by blurred vision, skin sensitization, and alteration in blood cell count [2].

Major metabolic reactions include hydroxylation of the indane ring, hydrolysis of the carboxymethyl group from the amino nitrogen, and opening of the oxadiazine ring, which gives rise to cleaved products. Indoxacarb had aromatic metabolites that can biotransform into active intermediates that produce methemoglobin [6]. Methemoglobin is a form of hemoglobin that has been oxidized, changing its heme iron configuration from the ferrous (Fe^2+^) to the ferric (Fe^3+^) state. The physiologic reduction of methemoglobin Hb Fe^3+^ to Hemoglobin (Hb) Fe^2+^ is mainly accomplished by red cell NADH cytochrome b5 reductase [3].

Unlike normal hemoglobin, methemoglobin does not bind oxygen, and as a result cannot deliver oxygen to the tissues. Normally, < 3% of hemoglobin is oxidized to methemoglobin each day, which is reduced by NADH methemoglobin reductase (cytochrome b5 reductase) and to a lesser extent by NADPH methemoglobin reductase, vitamin C, and glutathione enzyme systems [3]. Acquired methemoglobinemia results from exposure to drugs or toxins that cause oxidation of hemoglobin. When methemoglobin formation overwhelms protective enzyme systems, its levels rise and cause diminished oxygen delivery to tissues [7].

Contact with a poisonous substance can take place through ingestion, physical contact, translaminar action, during preening, and at rewetting of surfaces [8]. The typical presentation is of relative abrupt development of symptoms of hypoxia (low tissue oxygen) upon exposure to an oxidizing substance that induces methemoglobin formation. Cyanosis occurs with methemoglobin levels > 8–12%. Symptoms may range from cyanosis, dyspnea, or nonspecific symptoms (headache, lightheadedness, fatigue, irritability, and lethargy) to shock, severe respiratory depression, or neurologic deterioration (coma, seizures) due to tissue hypoxia, which can be fatal. Acquired methemoglobinemia is a medical emergency.

### Diagnostic clues

1. Exposure to toxin.
2. Cyanosis out of proportion to pulse oximetry.
3. Respiratory or neurologic symptoms.
4. Shock if severe dark red or brownish-to-blue blood that does not turn red with oxygenation.
5. Pulse oximetry of approximately 85% SaO2 that does not improve with oxygen.

Symptoms generally occur with methemoglobin levels > 10%, and levels > 30–40% can be life-threatening. Vitamin C is recommended for treatment of methemoglobinemia. It has been demonstrated that the reduction of the methemoglobin formation occurs at low vitamin C concentration in mice erythrocytes. Claro *et al*. showed that vitamin C (10–80 mmol/L^−1^) prevents the formation of methemoglobin by phenylhydrazone but did not have any effect on methemoglobin formation at the concentration of 90 mmol/L^−1^ [9].

Vitamin C has the potential to scavenge free radicals and protect cells from oxidative damage. Recycling of α-tocopherol by ascorbate has been demonstrated in cellular organelles and erythrocyte membranes. It also acts as a co-factor for NADP reductase required for glutathione metabolism. Furthermore, vitamin C can directly reduce methemoglobin and is proven to treat cyanosis effectively. Vitamin E is an antioxidant, protecting Red blood cell (RBC) from hemolysis induced through lipid per oxidation and the oxidation of sulfhydryl groups. Vitamin C and alpha-tocopherol in combination have been shown to have similar protecting effects on erythrocyte membranes exposed to an external oxidative stress [10].

The first‐line treatment of methemoglobin is intravenous methylene blue (MB), which acts as a co-factor to reduce the methemoglobin to oxyhemoglobin in the erythrocytes. Its potential side effects, such as methemoglobinemia and non-availability of methylene blue, restrict its usage in some special conditions. Vitamin C is a viable alternative drug with limited experience in methemoglobinemia. [11] In conclusion, this case reports shows a case of indoxacarb poisoning that presented with methemoglobinemia treated with vitamin C successfully to emphasize the use of vitamin C as an alternative method.

## Conclusion

Indoxacarb poisoning can be severe and potentially fatal. In this case, the patient ingested a compound containing indoxacarb in a suicidal attempt and initially showed no symptoms. However, patient was later found to have methemoglobinemia and low oxygen levels. The patient was treated with high doses of vitamin C, which is cost-effective and has a lesser side-effect profile as opposed to methylene blue. The treatment improved oxygen saturation and reduced methemoglobin levels. This case highlights the importance of early recognition and treatment of indoxacarb poisoning, as well as the potential benefits of using parenteral vitamin C in the management of methemoglobinemia.

## Data Availability

Available upon reasonable request from the corresponding author.

## Abbreviations

ABG: Arterial blood gas
OPC: Organophosphorus compound
SaO2: Oxygen saturation
ECG: Electrocardiogram
G6PD: Glucose-6-phosphate dehydrogenase
